# Coordination, framing and innovation: the political sophistication of public health advocates in Ireland

**DOI:** 10.1111/add.15404

**Published:** 2021-01-29

**Authors:** Matthew Lesch, Jim McCambridge

**Affiliations:** ^1^ University of York York UK

**Keywords:** Advocacy coalition framework, alcohol policy, coalition‐building, framing, learning, policy process, public health

## Abstract

**Background and Aims:**

This study explores the role of the public health advocacy coalition in alcohol policy development in Ireland. Compared with industry actors, much less is known about the membership, activities and influence of public health advocates in alcohol policymaking. To address this gap, this paper identifies several advocacy strategies, drawn from the advocacy coalition framework and other policy theories, and then analyses them in the context of recent Irish developments.

**Methods:**

The study used theory‐building process‐tracing to construct a record of the public health advocacy coalition and its campaign to promote the Public Health (Alcohol) Act 2018 in Ireland. Specifically, we drew upon 131 primary documents produced by advocates, 464 newspaper articles and 18 semi‐structured interviews with key advocates, public health experts and elected officials to undertake a thematic analysis.

**Results:**

Public health advocates in Ireland have developed sophisticated political strategies to foster major alcohol policy change. First, public health advocates led the formation of a broad‐based advocacy coalition that helped members to effectively pool their limited resources as well as coordinate their strategy and messaging. Secondly, issue‐framing and message discipline played a key role in the coalition's success. Advocates strategically focused upon the policy problem, specifically health harms, rather than the detailed content of the proposed measures. Finally, there is evidence of political learning, where advocates’ prior experiences and knowledge of the political system in Ireland spurred innovations in campaigning. These strategies were interdependent and mutually reinforcing, and succeeded in building support for public health advocates’ preferred policies among politicians and the general public.

**Discussion/conclusion:**

There are distinct capabilities that public health actors can mobilize in the policy process to win alcohol policy debates and capitalize on the constraints on industry influence on alcohol policymaking.

## Introduction

For more than two decades alcohol policy issues have been prominent in Ireland, with the growing recognition that alcohol‐related harms merited legislative responses [1]. Between 1984 and 2011, per‐capita alcohol consumption increased by more than 45% to levels which were very high by international standards [2]. The economic boom of the ‘Celtic Tiger’ years made alcohol more affordable in a culture where both patterns of heavy drinking and abstinence were well established [3]. The health consequences could be seen in the government's spending data from the 2000s, with an additional $3.7 billion allocated to address alcohol‐related harms every year [4].

Alcohol was first acknowledged as a health issue in the government's 1996 National Alcohol Policy, and again in two reports published by the Strategic Task Force on Alcohol in 2002 and 2004 [5]. A 2012 report prepared by the Steering Group on a National Substance Misuse Strategy (NSMS) linked rising consumption to increasing rates of alcohol‐related deaths, suicides and chronic illnesses [6]. Despite recognition of this problem, a legislative response was slow to develop.

A Fine Gael/Labour Coalition Government formed in 2011 after the deep impact of the global financial crash in Ireland and took action in October 2013, partly motivated by increasing health‐care costs. The Public Health (Alcohol) Bill was underpinned by four main pillars: (1) minimum unit pricing; (2) the structural separation of alcohol from other products in shops; (3) new restrictions on alcohol advertising and marketing, particularly as relating to children; and (4) new requirements for labelling [7]. The government's legislative proposals largely followed the Steering Group's policy recommendations which were, in turn, informed by the international scientific consensus on the evidence [8]. The structural separation and labelling provisions provoked the most resistance from the alcohol industry and its allies. After a protracted and contested process the legislation was signed into law in October 2018, with all four pillars approved by the legislature.

The alcohol industry and the public health community formed two opposing coalitions during the policy debate [9]. The stop–go nature of the legislative process followed duelling campaigns in the media and in parliament. The alcohol industry's success in resisting population‐level approaches to alcohol policy has been identified in Ireland [1, 5, 6, 10, 11, 12, 13, 14] and elsewhere [15, 16, 17, 18, 19, 20, 21, 22, 23]. In contrast, public health advocates have typically had limited success. Study of such advocacy groups is under‐developed globally, with few data available on membership, activities and impacts [24].

This study examines the public health advocacy community's efforts to promote the passage of the Public Health (Alcohol) Bill (hereafter, the Bill) in Ireland. Drawing upon concepts from policy studies [25] we identify different policy change mechanisms, including coalition‐building [26], issue‐framing [27] and political learning [9, 28] (see Table 1). We then analyse these factors in the context of the policy debate in Ireland.

**Table 1:** Possible theory‐based alcohol policy strategies for advocates

Tactics	Targets of advocacy groups	Description of mechanism and importance
Coalition‐building	Civil society groups	Enables collective action; allows for coordination of messaging and resources
	Elected officials and the public	Signalling device to policy actors about credibility/authority of coalition and widespread support
Issue‐framing	Elected officials and the public	Limits the scope of the debate to the coalition's preferred dimension of policy debate
Political learning	Elected officials and public	Draws on the distinct political expertise and experience of coalition members to identify successful tactics

Policy researchers have long been interested in the processes that facilitate major policy change [29]. The advocacy coalition framework (ACF) conceptualizes policymaking as a perennial struggle between competing coalitions. Advocacy coalitions consist of policy actors who ‘share a particular belief system’ and that ‘show a non‐trivial degree of coordinated activity’ [30]. Successful coalitions are those that translate beliefs into policy action [31, 32]. Enlarging the breadth and scope of a coalition's membership, or coalition‐building, is a key strategy for advocates [26, 33].

Issue‐framing presents a second mechanism for influencing the policy process [34, 35, 36]. Framing is a process that enables actors to ‘present their ideas’, ‘connect their ideas to important values’ and ‘persuade others of the validity of their particular interpretation of ideas’ [37]. Issue‐framing is intrinsically competitive where there are opposing coalitions [38].

Political learning shapes how advocacy coalitions pursue their goals. Studies of political learning [28] investigate how policy actors draw upon past experience and lessons from other jurisdictions. Scholars using the ACF find that ‘intra‐coalition learning’ is most likely to unfold when levels of inter‐coalition conflict are high [39].

Alcohol policymaking is characterized by a high degree of conflict between public health [40] and the alcohol industry [41]. Advocates, politicians, experts and government officials form the basis of public health coalitions. The latter three sets of actors tend to play secondary roles, finding ways to support advocates’ work and activities, without necessarily engaging in outward‐facing advocacy. The alcohol industry coalition consists of alcohol producers, wholesalers and retailers, as well as allies from other sectors and non‐health government departments.

Coalition members share a common ‘belief system’ [24] about the nature of the problem and preferred policy alternatives. Each coalition develops frames to broaden support for their positions [24, 41]. Public health actors (see Table 1), for instance, frame the harms associated with alcohol for society as a whole, while the alcohol industry typically emphasizes economic and personal responsibility frames [40, 41]. Broadening the focus to health and social impacts makes it easier to attract ‘new entrants’ into the public health coalition [42]. Conversely, the alcohol industry's focus upon the economic dimension of alcohol has helped to mobilize support from stakeholder groups with a potential interest in the sector's profitability, including specific government departments.

## Methods

The study describes the development and functioning of the public health advocacy coalition [43]. Drawing upon 131 primary documents (Table 2) produced by advocates and 464 newspaper articles (Table 3), we identified the key actors and issue frames. Primary documents were assembled by conducting on‐line searches of government websites, including the HRB's National Drugs Library. The Nexis database was used to collect relevant media published in three major national newspapers—*Irish Times*, *Irish Examiner* and *Irish Independent*—between 2012 and 2018.

**Table 2:** Alcohol advocacy communications analysed

Output type	Total records
Media communications (e.g. letters to editor and editorials) during policy debate (2012–18)^*^	24
Press releases during policy debate (2012–18)^**^	88
Policy documents (2008–2018)^***^	9
Total output	121

* Newspaper coverage included *Irish Times*, *Irish Examiner* and *Irish Independent*.

** This is not an exhaustive list of advocacy press releases; it only includes those produced by the Alcohol Action Ireland (AAI), the secretariat of the Alcohol Health Alliance Ireland. In some instances, member organizations released individual press releases, so this metric only captures a fraction of the total communication from advocates.

*** Policy documents were prepared either by advocates directly or by public/private organizations which had been commissioned to conduct the research.

**Table 3:** Newspaper coverage of alcohol advocacy actors 2012–18 (total *n* = 464)

Subject	Number of articles
Mention of	
Alcohol Action Ireland	81
Alcohol Health Alliance Ireland	18
Royal College of Physicians Ireland	66
Other key coalition members	43
Public health frame	325
Economic frame	169
Children and young people frame	211

Analytical propositions were triangulated with data from 18 semi‐structured interviews with advocates (12), senior politicians (four), and public health experts (two). The interviews were conducted in person or using Zoom between September 2019 and July 2020. Interviewees were purposively sampled and identified through documents, media and snowball sampling. E‐mail recruitment yielded a response rate of ~55%. The interviewees included several of the coalition's core members, including individuals from Alcohol Action Ireland (AAI) and the Royal College of Physicians Ireland (RCPI). Interviews were also conducted with representatives from several prominent national charities as well as the Irish Community Action on Alcohol Network.

Interviews were transcribed and uploaded to NVivo version 12 for thematic coding. Themes were generated both inductively (from the data, shaped by concepts from political science and policy studies) and deductively (from the concepts, and revising the framework, following review of the data) [41]. The transcripts were analysed iteratively, with both authors reviewing them and agreeing on interpretation.

## Results

The Public Health (Alcohol) Bill took approximately 5 years to be passed, and during this period was overseen by various health ministers. Divisions within the Coalition Government concerning whether the legislation should phase out alcohol industry sports sponsorship caused the first major delay [44]. After the planning and preparatory work, the Bill was formally introduced in December 2015, without the sports sponsorship proposals, but had little prospect of being adopted before the upcoming general election. The 2016 election saw both coalition partners suffer significant seat losses. Fine Gael subsequently formed a minority government, with a confidence and supply agreement with its fellow centre‐right party, Fianna Fáil, with support for its alcohol legislation [45]. In October 2016 the Bill was introduced in the Seanad (Senate, upper house). The structural separation provision prompted significant opposition, with retailers waging a major lobbying campaign and convincing several Fine Gael senators to delay progress [46]. The Bill's fortunes were revived in 2017, after Health Minister Leo Varadkar's elevation to Taoiseach (Prime Minister). The government then agreed to exempt smaller, rural shops from the regulation [47] and strengthened parts of the Bill, accepting an amendment from a cross‐party group to mandate the inclusion of cancer warnings as part of the Bill's labelling provisions [48]. The Bill proceeded to the Dáil (directly elected lower house), where warning labels dominated the debate. As the *Irish Times* remarked, politicians had ‘not been canvassed so assiduously since the smoking ban in 2004’ [49]. The legislation, however, was adopted in October 2018 with provisions due to be implemented over a 3‐year period.

### Coalition‐building

The public health alcohol advocacy community in Ireland had traditionally comprised a relatively small group of non‐governmental organizations (NGOs), public health experts, and public health officials. In the past two decades, however, Ireland has seen a concerted push to professionalize advocacy. AAI, a national advocacy organization, was formed in 2003 and participated in numerous policy deliberations, including the NSMS Steering Group. AAI particularly promoted alcohol pricing and availability measures, in line with the available evidence, in the 2000s. The organization also established relationships with Eurocare, an alcohol advocacy alliance at the European Union (EU) level and their member organizations in different countries. In 2013, when the government announced it would be acting on the Steering Group's recommendations, AAI mobilized a cross‐party group of senators and TDs (members of parliament).

Interest in alcohol policy also extended to the Irish medical establishment, leading to the formation of an alcohol policy group in the RCPI in 2012. As one advocate from the RCPI explained, ‘our [doctors] had been going to the international meetings of liver specialists and were alarmed by the rate of liver cirrhosis in Ireland’. Led by Dr Frank Murray, a liver specialist, the group hoped to inform alcohol policy by drawing on its members’ experience. This involved generating policy positions on population‐level measures [50], including minimum unit pricing and sports sponsorship [51].

Key figures in RCPI and AAI decided to mobilize other organizations who supported the principles of the Bill. Drawing inspiration from the contacts and organizational model of the UK counterpart, the Alcohol Health Alliance Ireland (hereafter, the Alliance) brought together 62 organizations, including numerous prominent health charities and professional medical bodies. The Alliance appointed Dr Frank Murray as Chair and a steering group to lead on strategy and communication. The Alliance was officially launched at RCPI in March 2015.

The resources and prestige associated with leading figures enhanced participation in the coalition. Many interviewees singled out Dr Murray and the RCPI, explaining how the two brought a ‘level of communications and PR expertise’ that the advocacy community had previously lacked. Others concurred, describing ‘the arrival of liver specialists’ as the game‐changer. This observation is consistent with research on advocacy from other settings. The growing interest in alcohol policy by liver doctors marked a major turning point in the mobilization of public health advocates in England [24].

Psychiatrists and public health specialists reinforced perceptions that the medical community viewed alcohol as a far‐reaching problem, impacting broadly on health and society. As one advocate explained, AAI had been ‘a small charity, a small voice, [and] a lone voice, with a limited budget’ and so the inclusion of organized medicine enabled the Alliance to ‘speak with more authority’ on alcohol harms. From a messaging perspective, the Alliance also assisted advocates in coordinating their activities. As one member remarked, traditionally there were ‘lots of disparate voices shouting out in an uncoordinated way’. The Alliance ensured that when an issue was raised in the media, the most effective voice articulated the public health position (see below).

Establishing a broad coalition also helped to strengthen the advocates’ credibility in the eyes of other actors. Although the steering group led on strategy, the optics of having a broad membership gave the Alliance ‘more clout with the public, with politicians, and with the media’. Supportive politicians who worked with the Alliance instructed them ‘to attach as many logos as possible’, as that would make it ‘impossible for [the politicians] to ignore’.

An additional advantage of the Alliance is that it allowed actors to draw ‘on the strength, and the reputations’ of its membership. Interviews revealed a systematic process for handling media inquiries, including an ‘information sheet’ about policy positions and a ‘contact sheet’ that was circulated to media outlets. The latter ensured that reporters were contacting the appropriate expert when they had questions about aspects of the Bill. For example, if queries arose concerning the link between alcohol and cancer, the media would be directed to the Irish Cancer Society. As one explained: The coalition was broader than the formal alliance, however, as it developed close working relationships with politicians and key officials within the health department. For example, several politicians became champions of the Bill at different junctures and Dr Tony Holohan, the Chief Medical Officer, was a prominent and long‐standing supporter. These relationships allowed the Alliance to ‘[keep] on top of what was happening in government… and what… might have been said in the media’. Moreover, to maintain message discipline, the steering group advised experts ahead of their media appearances. The Alliance also recruited individuals from member organizations who possessed a background in public affairs, including those with prior roles as journalists and as political advisers, thus possessing an intimate knowledge of the political system (see below).

### Issue‐framing

Issue‐framing played a key role in establishing the policy debate's key contours. The Alliance used its website and other communication materials to highlight the harms caused by high levels of alcohol consumption. Advocates also released hundreds of documents, including reports and press releases, to generate media coverage (see Table 2). Advocates also used social media, press interviews and editorials at key stages of the Bill's progression. When the Bill was first published in spring 2015, a collection of editorials were written to underline the principal harms frame [52, 53, 54, 55].

The Alliance's media presence afforded key opportunities to underline the extent of alcohol‐related harms in Ireland. Although the alcohol industry's focus upon the economic impact of the Bill (i.e. economic framing) was also evident in the newspaper coverage (36% of articles), coverage of health harms and the effects of alcohol on children and young people were more prominent (in 70 and 45% of articles, respectively).

The Alliance engaged in lobbying, although it faced entrenched alcohol industry positioning, and found clever ways to pivot when confronting less advantageous frames. When industry identified the economic costs of the Bill, advocates responded by highlighting the health‐care costs associated with the *status quo*. This resonated, as concerns over ‘wait times, delayed discharges and limited beds’ were common in the media. ‘The Health Service was creaking under the weight of alcohol… and we used every opportunity to highlight that’, one advocate explained. Health‐care issues were a key voter concern in the 2016 general election (and remained so in the subsequent 2020 general election).

A key framing strategy was to focus upon the content of the problem—the health harms—rather than the particular measures within the Bill itself. As one strategist explained:

My advice to the doctors when they were going on radio was ‘you need to make this an everyday issue for people to understand this… you need to give examples of the likes of people who you are seeing’.

The structural separation debate underscored the risks in debating policy solutions outside health or their implementation, and which mobilized actors in the broad industry coalition. While health experts could credibly speak about their experience treating alcohol‐related harms, they were less adept at discussing practical implementation issues in non‐health settings.

Advocates made extensive use of social media and developed multi‐media strategies to establish their preferred framing. For example, photographs were posted with questions underneath, such as: ‘if your child goes into the shop, why are they looking at a bottle of Vodka beside a packet of Smarties?’ Mental health issues were salient in the media in the years running up to the Bill. Alcohol contributed to half of all male suicides in Ireland, which were very high by international standards. AAI produced a short video to bring this home, featuring John Higgins's story, a man who had lost his son, David, to suicide. In the video, David's death is attributed to the availability of cheap alcohol. The video garnered more than 60 000 views on YouTube.

Additionally, the Alliance used infographics to help the public visualize the damage of alcohol‐related harms. Three deaths every day could be attributed to alcohol, and this statistic was underlined in every press release and infographic. AAI's press releases would estimate mortalities occurring since the Bill was first introduced (and similarly supplied political representatives with constituency‐level data; see below). For example, in May 2018, AAI issued the following release:

This week… marks the passing of 900 days since the [Bill] began its legislative passage… Over that time sadly the shocking levels of alcohol‐related harms have continued unabated: 2700 deaths were alcohol‐related [56].

Evidence suggesting the Alliance's campaigning was highly successful is not limited to interview data. Advocates were highly visible in newspaper coverage, garnering specific mentions in 36% of newspaper articles about the Bill. Alliance members also wrote many letters to the editor. As one advocate explained:

If [we] had a letter in the paper… it was easier to get a meeting on that day [with politicians]… so the media campaign [supported] the advocacy work and vice‐versa [57].

Framing activities thus facilitated access to key actors. The Alliance's efforts were also recognized by non‐public health specialists. The campaign won an award for Best Public Affairs Campaign by two national public relations organizations. According to one citation, the Alliance's ‘expert stakeholder alliances and evidence‐based communications’ succeeded in keeping alcohol on the agenda for more than 3 years, despite facing a ‘well‐resourced and culturally‐embedded opposition’ [57].

### Political learning

Convincing politicians to support policy change poses obvious challenges for advocacy groups, even in this context, where none of the political parties opposed the legislation. We found that advocates possessed specific expertise that was critical to the Bill's passage. Of the advocates interviewed, a majority had some previous experience in the formal sphere of politics (e.g. former politician or policy adviser) or journalism. Some key figures, such as Suzanne Costello, the CEO of AAI, had backgrounds in campaigning organizations. These experiences provided advocates with heightened capabilities for political strategy. The Alliance formed close relationships with sympathetic politicians, thus broadening the coalition, and leveraging these interactions to shape the legislative process. As one former senator recalled: The nature of the relationships between Alliance members and individual politicians beyond the coalition varied greatly. In some instances, advocates found themselves meeting with ministers about something unrelated to the Bill but were encouraged to raise the Bill's profile. The Alliance's lobbying efforts also took on more direct forms. For example, the Alliance organized several information sessions at the Oireachtas (Parliament) concerning alcohol‐related harms. As one expert explained, the meetings allowed politicians to ‘pick our brains’.

Others described key lessons that had been drawn from other successful public health campaigns in Ireland, including the ban on smoking in enclosed public places. According to one politician, ‘nobody gave [the smoking bill] a chance given the strength of the publican's lobby… but by “mobilising an alliance of unlikely advocates [such as] academics… and organised labour” the advocacy coalition served as an effective counterweight to the industry and its allies’.

Advocates also harnessed political information to target politicians who were sympathetic to industry efforts to pick apart the legislation. The Alliance sent constituency‐specific information to draw politicians’ attention to the total number of alcohol‐related deaths in their communities. Local grassroots organizations played a key role in reinforcing this tactic at the local level. Constituents were encouraged to write letters and send e‐mails to their TDs: Alliance members used their knowledge of parliamentary processes and contacts. The Alliance, for example, mobilized and assisted politicians on a cross‐party basis to propose amendments, including by formally establishing a group. Advocates believed that they needed to be ‘proactive and on the offensive’. Introducing amendments that strengthened the Bill ‘rather than waiting for the industry to weaken its existing parts’ was a key tactic. As one advocate explained, ‘if you give [politicians] the amendments… it makes it easier and more likely that they will take it upon them, and… table them’. Although many amendments were rejected, the Alliance secured a key victory when the government amended the legislation to include cancer warning labels.

Finally, advocates found a clever way to publicize the extent of industry lobbying. In 2015, a lobbying registry took effect in Ireland, requiring all lobbying activities to be publicly disclosed. This was largely instigated by the Labour Party in the Coalition Government, who have also been the early champions of the Bill. Information from the registry was then made public. Key coalition members realized the potential of this new resource. As one advocate explained: In several instances, reporters from the *Irish Times* published stories about the industry's lobbying efforts [46, 49, 58, 59, 60, 61, 62].

Advocates exhibited sophistication throughout the debates over the Bill. One expert, who was not formally involved in the coalition, had this to say about its influence:

[The Alliance] was pivotal because they made it a campaign and they ran it like a campaign… Campaigns are not accidental things… they have to have many arms… they have to have a communications arm, a political arm, a policy arm, [and] a civil service arm… I think they covered all the bases… I think the public health side [was] strong and… more savvy than before.

## Discussion

Our analysis documents the growing political sophistication and effectiveness of public health advocates in Ireland. First, a broad‐based coalition enabled advocates to pool resources and coordinate their strategy and messaging. Secondly, issue‐framing was critical in shifting the focus of the debate to alcohol‐related harm. This placed pressure upon politicians by making available evidence on the extent of the problem. Finally, we present evidence of political learning, where advocates’ knowledge of the political system spurred innovations in campaigning.

The three mechanisms identified in our analysis are distinct but also inter‐related. Creating a broad‐based coalition reflected lessons drawn from previous experience with the Smoking Bill. Furthermore, the public health framing mattered precisely because the Alliance's architects had mobilized credible experts and organizations. Thus, the effectiveness of each component was predicated on synergies with other elements of strategy. Our findings provide insights into the developing capacity of advocates to drive major policy change.

This study contributes to a sparse literature [24, 40, 63]. Among the study limitations are the challenges of using recall of activities, spanning more than 5 years in some cases, and interrogating actors’ opinions. These data, however, have been triangulated with data from other sources in making inferences.

In considering the adoption of world‐leading alcohol policy innovation in Ireland, the specific context of major cultural and political change over the past three decades should also be recognized. The country's early adoption of the ban on smoking in public places, as well as national referenda on divorce, abortion and same‐sex marriage and subsequent change, resulted from hard‐fought advocacy campaigns. Such successes generate momentum and are relevant to appreciating how the broader advocacy community have professionalized over time.

Ireland's long‐standing problematic relationship with alcohol as well its failure to get to grips with the problem is another important consideration [1, 5, 6, 11]. Interviewees testified to the foundational research and/or advocacy of key individuals, including Ann Hope, Shane Butler and Joe Barry (who has continued in a leading role), in interpreting international evidence in the Irish context. These experts were instrumental in generating high‐level awareness of the existence of a problem, gradually re‐framing the problem away from an issue affecting a subgroup of ‘alcoholics’ towards a fuller population‐level understanding of alcohol harms. The historical roots of policy innovation in Ireland were not the focus of our analysis [6] but the significance of such efforts is clearly relevant. Politicians were receptive to major policy change due in part to sustained public attention to alcohol as a problem [11].

This study contributes to a broader research programme on the alcohol industry and the role of evidence in alcohol policymaking [21, 41, 64, 65]. A recurring finding on the alcohol industry lies in the advantages it holds over its opponents with respect to resources and lobbying efforts [41]. This study, however, shows that experts often possess unique capabilities or attributes—such as public trust—which can help to mitigate the industry's resource advantage. Future research could consider whether and how far the consumption of policy information (e.g. frames) is subject to source effects [27] and the extent to which trust can mediate this relationship [66].

More broadly, our study illustrates how better engagement between policy analysis and alcohol research can generate insights for both research traditions. Policy theory can help alcohol researchers to identify the mechanisms underlying policy inertia and change, including coalition‐building and political learning [25]. This particular study also shows how alcohol policy developments can inform policy theory. In the case of the ACF, the framework's core hypotheses have found support across a range of settings [24, 67]. Coordination and collective action have been the least‐studied parts of the framework [68]. This study suggests that there are potential links between different mechanisms—coordination and intra‐coalition learning—in the framework.

Finally, the prolonged development of alcohol policy innovation in Ireland underscores the perennial role of conflict. The Irish advocacy coalition saw itself as fighting a war with industry in pursuit of rational policymaking, based on using high‐quality scientific evidence to reduce avoidable harms caused by alcohol. In such terms, an important series of battles have been won, culminating in the passage of what has become the Public Health (Alcohol) Act 2018. However, the political war will not end with legislative enactment. Researchers will need to focus upon policy implementation, examining how each coalition seeks to advance its interests and ideas in this next stage of the policy process.

## Declaration of interests

None.

## Author contributions

**Matthew Lesch:** Conceptualization; data curation; formal analysis; investigation; methodology; project administration; software. **Jim McCambridge:** Conceptualization; data curation; formal analysis; funding acquisition; investigation; methodology; project administration; supervision.
